# Oxygen physiology: sensors and ion channels

**DOI:** 10.1007/s00424-015-1762-9

**Published:** 2015-11-21

**Authors:** Yasuo Mori, Nobuaki Takahashi, Nozomi Ogawa, Thomas Gudermann

**Affiliations:** Laboratory of Molecular Biology, Department of Synthetic Chemistry and Biological Chemistry, Graduate School of Engineering, Kyoto University, Kyoto, 615-8510 Japan; Laboratory of Environmental Systems Biology, Department of Technology and Ecology, Hall of Global Environmental Studies, Kyoto University, Nishikyo-ku, Kyoto, 615-8510 Japan; Department of Cell Biology, Harvard Medical School, Boston, MA 02115 USA; Walther-Straub-Institute for Pharmacology and Toxicology, Ludwig-Maximilians University, Munich, Germany; Comprehensive Pneumology Center Munich (CPC-M), German Center for Lung Research, Munich, Germany; DZHK (German Centre for Cardiovascular Research), Munich Heart Alliance, Munich, Germany

Ever since Pflüger, it has been always a major issue of physiology to identify the core principles underlying our body’s ability to sense and adapt to the changes in the availability of ambient molecular oxygen (O_2_) [3, 5]. The milestone discovery of an O_2_ sensor was first achieved at the organ level by Heymans, who demonstrated that inspired hypoxia triggers increased breathing through the stimulation of the carotid bodies (CBs) [2]. With regard to the cellular and molecular mechanisms underlying the acute hypoxia-sensing in the CBs, it is generally accepted that hypoxia inhibits K^+^ channels to depolarize the chemoreceptor glomus cells, leading to the activation of voltage-dependent Ca^2+^ channels and consequent release of neurotransmitters that relays information to regulate the respiration [10]. Another milestone is the discovery of a soluble factor erythropoietin (Epo) that is essential for the chronic phases of O_2_ adaptation. At the end of nineteenth century, it was recognized by Viault that exposure to high altitude hypoxia elicits a robust burst of erythropoiesis in humans [1]. Almost a half century later, Epo was demonstrated to mediate hypoxia-induced erythropoiesis. While the initial characterizations of the expression of Epo suggested its production in the fetal liver and adult kidney, it was only recently that the localization of the renal Epo-producing (REP) cell was found to be in the peritubular interstitial space located in the deep renal outer medulla region [4]. In addition, hypoxia-inducible factor (HIF) [6] is recognized as the transcription factor central to chronic cellular responses to hypoxia including erythropoiesis via Epo production. Thus, CBs, Epo, and HIF provide solid bases for understanding the body’s ability to sense and adapt to the ambient O_2_.

Even after the above discoveries of the core principles, the physiology of O_2_ sensing and adaptation never loses its glory. This is highlighted by recent findings concerning the diversity and ubiquity of O_2_-sensing mechanisms. For example, in addition to multiple K^+^ channel subtypes and mediators previously reported responsible for hypoxia sensing, transient receptor potential (TRP) cation channels have emerged as O_2_–regulated ion channels critical for O_2_ sensing in non-CB chemoreceptors and tissues [7, 11]. Importantly, HIF, which binds to the hypoxia-responsive element present in the 3′ enhancer region of the Epo gene [6], is expressed widely throughout the body and is intriguingly found in lower organisms that produce neither Epo nor red blood cells. In addition to erythropoiesis, chronic hypoxia induces the expression of numerous genes whose products facilitate non-oxidative synthesis of ATP and multiply the number of vessels supplying the hypoxic area of tissues [8] through the action of HIFs. These lines of evidence suggest the ubiquitous presence of O_2_-sensing cells and organs in the animal body. In this context, it is interesting to note that two HIF isoforms HIF1 and HIF2 play distinctive roles in the regulation of hypoxic responses of CBs by differentially promoting cellular redox status [12]. Moreover, the physiological importance of hypoxia has been demonstrated in maintaining the hematopoietic stem cell niche for cell cycle quiescence through the action of HIF in the bone marrow [9]. In this special issue, reviews of these recent developments are included together with several important original research articles, which indeed enrich our knowledge in O_2_ physiology.

We believe that there is a great prospect that O_2_ physiology will further enhance its current status as a major issue in physiology. A future direction of O_2_ physiology is systems physiology, in which regulation of O_2_ sensing and adaptation through interplays among different chemoreceptors and molecules are studied. Another possible direction is to understand O_2_ sensing and adaptation in the context of redox biology, where alterations in redox homeostasis (via reactive chemical species and antioxidant systems) in response to changes in the O_2_ availability are integrated with other types of stress such as metabolic perturbation, heat, and proinflammatory mediators. Adding these future developments to O_2_ physiology should lead us to understand our body’s ability as a total system to adapt to changes in ambient O_2_ availability.
